# Interstitial photodynamic therapy for newly diagnosed glioblastoma

**DOI:** 10.1007/s11060-023-04284-9

**Published:** 2023-03-16

**Authors:** Stefanie Quach, Christoph Schwartz, Maximilian Aumiller, Marco Foglar, Michael Schmutzer, Sophie Katzendobler, Mohamed El Fahim, Robert Forbrig, Katja Bochmann, Rupert Egensperger, Ronald Sroka, Herbert Stepp, Adrian Rühm, Niklas Thon

**Affiliations:** 1 Department of Neurosurgery, University Hospital Munich, Ludwig-Maximilians-University, Marchioninistrasse 15, 81377 Munich, Germany; 2 Department of Neurosurgery, University Hospital Salzburg, Paracelsus Medical University Salzburg, Salzburg, Austria; 3 Laser-Forschungslabor, LIFE Center, University Hospital Munich, Ludwig-Maximilians-University, Planegg, Germany; 4 Department of Urology, University Hospital Munich, Ludwig-Maximilians-University, Munich, Germany; 5 Department of Neuroradiology, University Hospital Munich, Ludwig-Maximilians-University, Munich, Germany; 6 Max Planck Institute of Psychiatry, Munich, Germany; 7 Center for Neuropathology and Prion Research, University Hospital, LMU Munich, 81377 Munich, Germany; 8 German Cancer Consortium (DKTK), Partner Site Munich, Munich, Germany

**Keywords:** 5-aminolevulinic acid, Interstitial photodynamic therapy, Glioblastoma, Overall survival, Postoperative morbidity, Progression-free survival

## Abstract

**Purpose:**

Innovative, efficient treatments are desperately needed for people with glioblastoma (GBM).

**Methods:**

Sixteen patients (median age 65.8 years) with newly diagnosed, small-sized, not safely resectable supratentorial GBM underwent interstitial photodynamic therapy (iPDT) as upfront eradicating local therapy followed by standard chemoradiation. 5-aminolevulinic acid (5-ALA) induced protoporphyrin IX was used as the photosensitizer. The tumors were irradiated with light at 635 nm wavelength via stereotactically implanted cylindrical diffuser fibers. Outcome after iPDT was retrospectively compared with a positively-selected in-house patient cohort (n = 110) who underwent complete tumor resection followed by chemoradiation.

**Results:**

Median progression-free survival (PFS) was 16.4 months, and median overall survival (OS) was 28.0 months. Seven patients (43.8%) experienced long-term PFS > 24 months. Median follow-up was 113.9 months for the survivors. Univariate regression revealed MGMT-promoter methylation but not age as a prognostic factor for both OS (p = 0.04 and p = 0.07) and PFS (p = 0.04 and p = 0.67). Permanent iPDT-associated morbidity was seen in one iPDT patient (6.3%). Patients treated with iPDT experienced superior PFS and OS compared to patients who underwent complete tumor removal (p < 0.01 and p = 0.01, respectively). The rate of long-term PFS was higher in iPDT-treated patients (43.8% vs. 8.9%, p < 0.01).

**Conclusion:**

iPDT is a feasible treatment concept and might be associated with long-term PFS in a subgroup of GBM patients, potentially via induction of so far unknown immunological tumor-controlling processes.

## Introduction

Glioblastoma (GBM) is the most common malignant brain tumor [1]. Current treatment concepts comprise maximal safe resection followed by a combination of radiotherapy and chemotherapy with temozolomide [2], possibly augmented by tumor-treating fields [3]. Despite this aggressive treatment regimen, median survival is limited to 15–20 months [2–4]. Unfavorable outcome must be particularly expected if the tumor cannot be resected completely due to eloquent location [5, 6], and/or if an unmethylated O6-methylguanin-DNA-methyltransferase (*MGMT*) promoter hampers response to chemotherapy [7]. Thus, alternative treatment concepts need to be evaluated.

Photodynamic therapy (PDT) is a local treatment concept used for a variety of neoplastic [8, 9] and non-neoplastic conditions [10]. It is based on the light-induced activation of a photosensitizer leading to the formation of reactive oxygen species and subsequent apoptosis and necrosis of the affected cells [11]. The photosensitizer protoporphyrin IX is preferentially synthesized within malignant glioma cells after oral application of its precursor 5-aminolevulinic acid (5-ALA). This highly specific accumulation makes 5-ALA a well-suited photosensitizer predrug for PDT [12]. The good tumor-to-background-ratio of protoporphyrin IX synthesis is regularly exploited in fluorescence-guided resection [13]. For tumors unamenable to safe complete resection, interstitial PDT (iPDT) has been explored as a minimally invasive procedure where treatment light is applied through stereotactically implanted optical fibers. IPDT was found to be a feasible salvage treatment option in small malignant glioma case series [14–16]. Recently, a larger series of recurrent malignant gliomas reported a post-recurrence survival longer than 24 months for 25% of the treated patients [17].

Based on these promising results in GBM recurrences, we offered iPDT as an alternative local treatment option upon specific demand to patients with small-sized, unifocal, not safely resectable, newly diagnosed GBM. In here, we share our experiences and outcome data, focusing on progression-free survival (PFS), overall survival (OS), and treatment-associated morbidity in a series of 16 adult patients with untreated GBMs undergoing iPDT as primary treatment. All patients received postoperative standard treatment with radiation therapy plus concomitant and adjuvant temozolomide. Outcome data after iPDT were put into perspective with an in-house cohort of GBM patients having undergone complete tumor resection followed by a complete course of radiochemotherapy according to the EORTC/NCIC protocol [2].

## Patients and methods

### IPDT patient cohort

All patients were discussed in advance in our local interdisciplinary neurooncological tumor board. The decision to perform iPDT in selected cases was triggered by the patients’ specific demand as well as our prior experiences with iPDT treated malignant glioma recurrences. Eligibility criteria for patients undergoing iPDT on specific demand consisted of (1) small-sized (diameter < 4 cm), circumscribed, untreated GBMs without or moderate midline shift without signs of transtentorial herniation or contact to the ventricular system, (2) unifocal, supratentorial, and (3) patients should rate on the Karnofsky performance scale (KPS) with values ≥ 70. All patients were informed in detail about the procedure and its associated risks, and about the fact that iPDT is not the established standard treatment for newly diagnosed GBMs and is considered an individual treatment attempt. Written informed consent was obtained from all patients. The institutional review board approved the protocol for the retrospective analysis (ethics approval no. 335 − 16, Ludwig-Maximilians-University, Munich, Germany).

### Study cohort for comparative analyses

An in-house patient cohort was used for comparative outcome analyses. This cohort included 110 highly selected patients who had received the optimal available first-line treatment for newly-diagnosed GBMs consisting of complete resection (as proven by early post-operative MRI) followed by a full course of adjuvant radiochemotherapy according to the EORTC/NCIC protocol.

### Treatment procedure

**Fig. 1:**
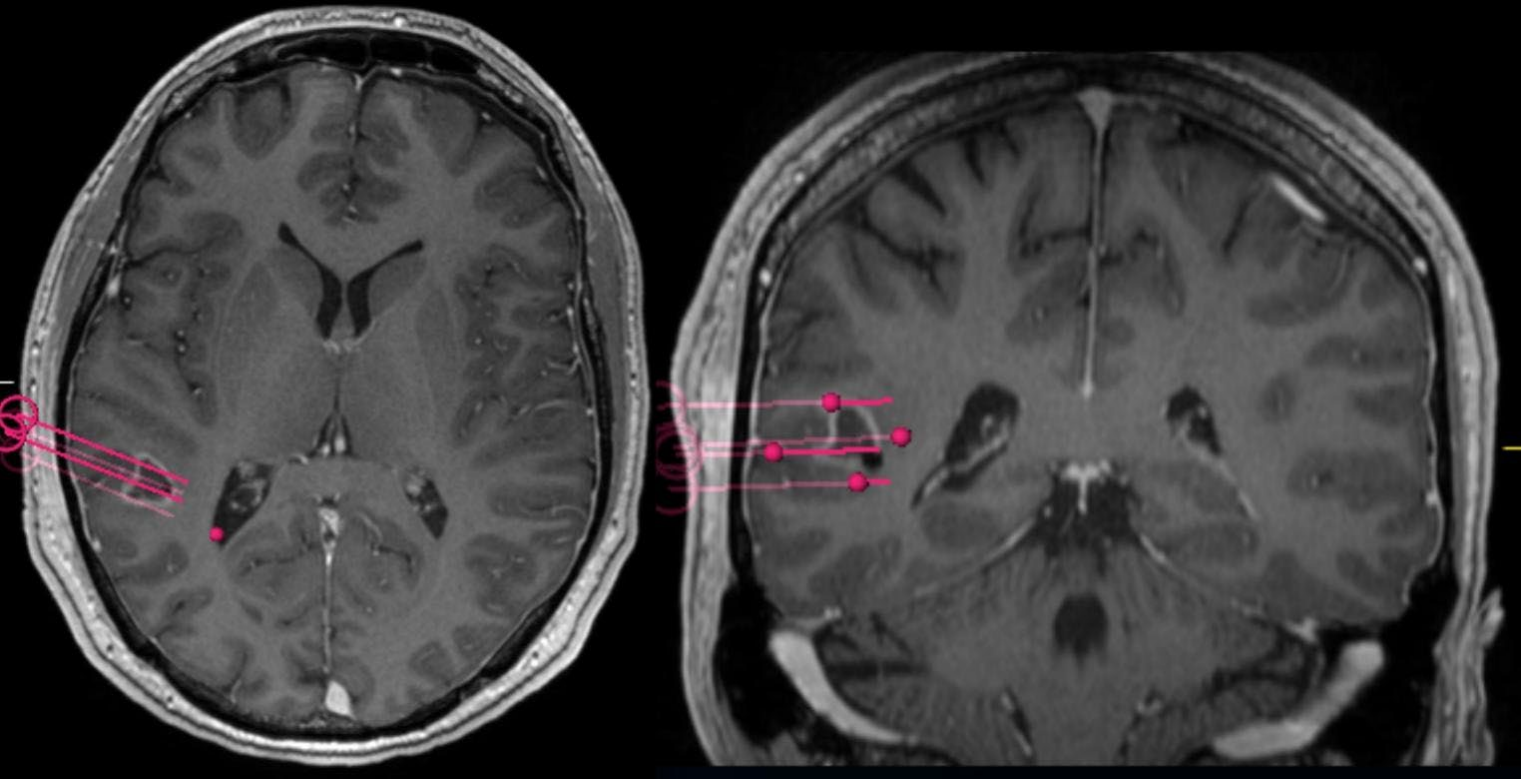
Exemplary case illustrating three-dimensional planning of the light diffusor trajectories. The patient (IPDT 02) presented with seizures and aphasia leading to the diagnosis of glioblastoma in the left angular gyrus

All tumors were diagnosed histologically according to the current WHO classification at the time of treatment [18]. *MGMT* methylation status, isocitrate dehydrogenase (*IDH)* mutation status and LOH1p/19q were determined as described previously [19]. Interstitial photodynamic therapy was performed in a standardized fashion as reported in detail before [14, 17]. In brief, a three-dimensional treatment volume was defined using preoperative MRI with contrast-enhanced T1 and, when available, O-2-[^18^ F]fluoroethyl-L-tyrosine-positron emission tomography (FET-PET). These images, together with T2-weighted images and contrast-enhanced MR-angiography, were fused to the intraoperatively acquired stereotactic computerized tomography (CT) to plan the trajectories of the cylindrical light diffusors (CYD 600, Light Guide Optics, Rheinbach, Germany). Figure 1 shows an implantation schematic. Three hours after systemically administering 5-ALA (medac GmbH, Wedel, Germany) at a standard dose of 20 mg/kg bodyweight (maximum: 30 mg/kg), the light diffusors were implanted stereotactically under general anesthesia. Light irradiation was performed at a wavelength of 635 nm (median total dose: 12,240 J, range 7200–20,520 J; median dose per treatment volume: 2.400 J/cm^3^, range 969–5760 J/cm^3^; median light power per diffusor length: 200 mW/cm, range: 100–200 mW/cm; Ceralas PDT Diode Laser, biolitec AG, Jena, Germany). The median duration of irradiation was 1.0 h (range: 1.0 to 2.0 h, elongated irradiation times were compensated by a reduced radiant flux). Prior to and after the irradiation, intraoperative spectral online monitoring measurements were performed to document the transmission of the treatment light through the tissue and the amount of PpIX fluorescence generated therein as well as to monitor for potential treatment-related or treatment-relevant effects [20, 21].

### Adjuvant treatment and follow-up

In accordance with Stupp protocol [2], all but one patient received concomitant radiochemotherapy and adjuvant cycles of temozolomide chemotherapy. One patient (IPDT 11) was treated by adjuvant radiotherapy only because of an unmethylated *MGMT*-promoter status in combination with advanced age > 65 years. Adjuvant treatment was initiated within two weeks after iPDT in all patients. Any treatment-associated morbidity was documented. The last clinical follow-up for this specific cohort was January 12th, 2022.

### Evaluation and statistical analyses

The two study endpoints, PFS and OS, were calculated from the date of iPDT or tumor resection (reference cohort). Long-term survival was defined as a PFS of > 24.0 months as calculated by Kaplan-Meier method. Follow-up was assessed by the reverse Kaplan-Meier method. Survival was evaluated with the Kaplan-Meier method and compared by the log-rank test. As potential prognostic factors, *MGMT* methylation status and age were assessed by univariate regression because of the lesser number of events in the iPDT cohort. Categorical variables were analyzed using the χ^2^ test and age with Student’s t-test. A p-value ≤ 0.05 was considered significant. All calculations were performed using SPSS Statistics 25 (IBM, Armonk, New York, USA).

## Results

### Patient population

Sixteen patients were consecutively treated with iPDT between 2008 and 2014. Their median age at iPDT was 65.8 years (range 29.7–76.5), and all had a KPS of 90. Clinically, epilepsy (n = 12), aphasia (n = 3), and hemiparesis (n = 1) led to the tumor diagnosis. The treated tumors were localized in the temporal lobe (n = 8), the frontal lobe (n = 3), the parietal lobe (n = 4), and central gyrus and subcentral lobe (n = 1). Fourteen tumors were located in the dominant hemisphere. A methylated *MGMT*-promoter status was seen in 8/16 (50.0%) and an *IDH* mutation in 2/16 (12.5%) of iPDT patients. No tumor had a 1p/19q codeletion (LOH1p/19q). The median tumor volume was 6.1 cm^3^ (range: 1.4–21.8 cm^3^); the number of implanted light diffusors ranged from three to ten per tumor. Table 1 details histopathological profiles at the time of iPDT and outcome data for the iPDT patient cohort.

**Table 1:** Patient characteristics including biomarker status and follow-up

Patient number	sex	Age at iPDT* (years)	*MGMT* promoter methylated	*IDH* mutation	Ki67 proliferation index	PFS (months)	OS (months)	status
IPDT 01	m	29,7	yes	yes	?	64,7	102,4	deceased
IPDT 02	m	40,6	no	no	10%	59,2	95,0	deceased
IPDT 03	f	50,3	yes	no	10%	127,1	127,5	alive
IPDT 04	m	69,9	no	no	30%	8,3	15,0	deceased
IPDT 05	m	68,2	no	no	15%	12,0	16,1	deceased
IPDT 06	m	63,7	no	no	20%	4,3	9,0	deceased
IPDT 07	m	70,1	yes	no	25%	110,1	110,3	alive
IPDT 08	f	74,1	no	no	25%	60,6	66,4	deceased
IPDT 09	m	33,3	partially	yes	85%	113,6	113,9	alive
IPDT 10	f	74,3	no	no	30%	16,4	28,0	deceased
IPDT 11	m	68,8	no	no	21%	6,0	8,5	deceased
IPDT 12	m	68,0	yes	no	7%	6,5	8,0	deceased
IPDT 13	f	57,3	partially	no	15%	9,5	25,2	deceased
IPDT 14	m	54,3	yes	no	15%	35,7	43,9	deceased
IPDT 15	m	76,5	no	no	10%	7,4	9,2	deceased
IPDT 16	m	53,4	yes	no	28%	17,8	36,4	deceased

* iPDT = interstitial photodynamic therapy; *MGMT* = O6-methylguanin-DNA-methyltransferase; *IDH* = isocitrate dehydrogenase; PFS = progression-free survival; OS = overall survival; m = male; f = female.

MGMT promoter methylation status was determined as described before [22].

### Survival after iPDT

**Fig. 2:**
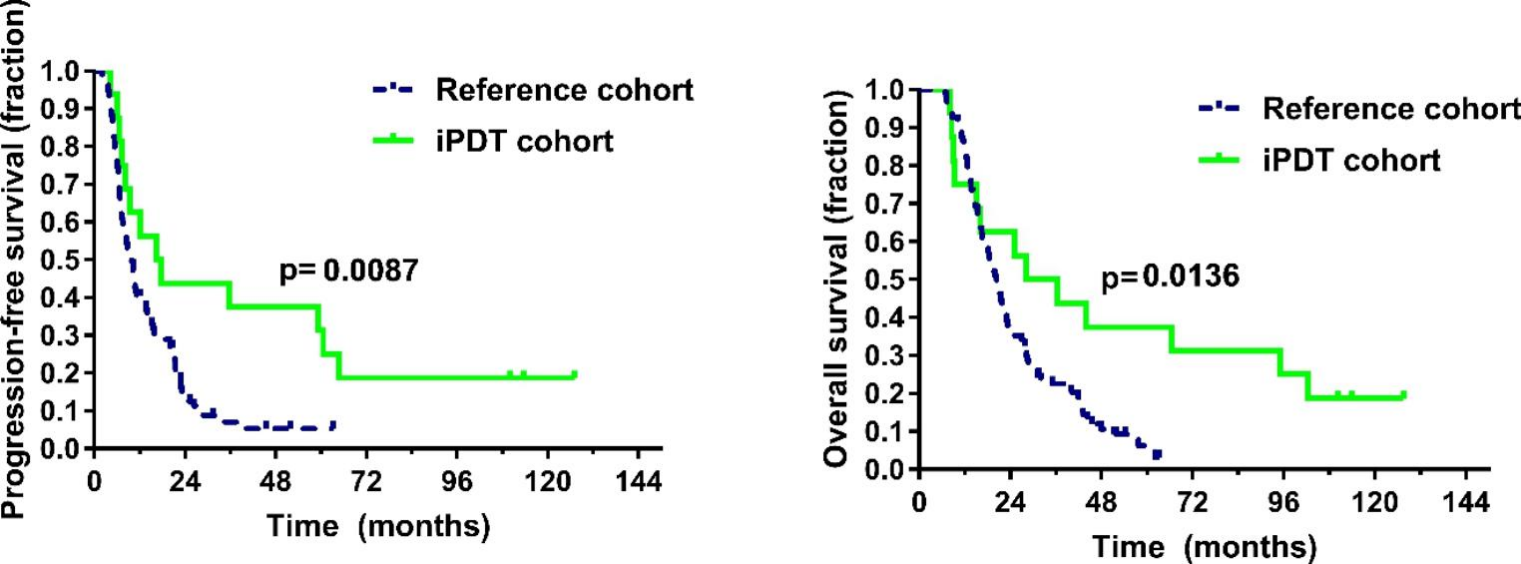
Progression-free survival and overall survival were significantly longer in the interstitial photodynamic therapy (iPDT) cohort compared to the reference cohort

Median follow-up for the survivors was 113.9 months. Within this follow-up, 13 patients succumbed to their disease. Median PFS was 16.4 months; median OS was 28.0 months (see Fig. 2). One-year and two-year PFS rates were 56.3% and 43.8%, one-year and two-year OS rates were 75.0% and 62.5%, respectively. Univariate regression revealed *MGMT*-promoter methylation but not age as a prognostic factor for both OS (p = 0.04 and p = 0.07) and PFS (p = 0.04 and p = 0.67). At the time of recurrence, the patients received bevacizumab/irinotecan (n = 5), re-radiotherapy (n = 3), brachytherapy (n = 3), temozolomide rechallenge (n = 3), procarbazine/lomustine chemotherapy (n = 2) or a combination thereof. Four patients presented with cognitive deterioration at first recurrence and received best supportive care; no patient underwent open tumor resection.

### Comparison to reference population

Patients in the reference cohort resembled iPDT patients with respect to age (median 56.1 years (range: 17.2–86.6 years) vs. 65.9 years, p = 0.21), *MGMT* promoter methylation (methylation rate: 48/99 (48.5%) vs. 8/16 (50.0%), p = 0.91), and *IDH* mutation rate (3/40 (7.5%) vs. 2/16 (12.5%), p = 0.55). KPS was higher in the iPDT treated group (90 for all iPDT patients, median 80, range 60–90 in the reference cohort, p < 0.01). *MGMT* methylation, but not age were associated with a prolonged OS (p < 0.01 and p = 0.12) and PFS (p < 0.01 and p = 0.07).

Patients treated with iPDT experienced superior progression-free and overall survival compared to patients who had undergone complete tumor resection (p < 0.01 and p = 0.01, respectively). The rate of patients with long-term PFS (> 24 months) was higher in iPDT treated patients (43.8% vs. 8.9%, p < 0.01, see also Table 2).

Among patients with *MGMT*-methylated tumors, 62.5% of iPDT treated patients experienced long-term PFS (> 24 months), compared to 16.7% in the reference group (p < 0.01).

**Table 2:** Comparison between iPDT and reference cohort

		iPDT cohort	Reference cohort	p-value
Median age (range)		65.8 years (29.7–76.5 years)	56.1 years (17.2–86.6 years)	0.21
*MGMT* promoter methylated		8/16 50.0%	48/99* 48.5%	0.91
*IDH* mutant		2/16 12.5%	3/40* 7.5%	0.55
Median PFS (95% CI)		16.4 months	9.9 months	< 0.01
Median OS (95% CI)		28.0 months (6.0–50.0)	20.4 months (17.9–22.9)	0.01
Long-term PFS (> 24 months)		7/16 (43.8%)	11/110 (8.9%)	< 0.01

* Molecular analyses were unavailable for some patients in the reference cohort.

*MGMT* = O6-methylguanin-DNA-methyltransferase; *IDH* = isocitrate dehydrogenase; PFS = progression-free survival; CI = confidence interval; OS = overall survival.

### Postoperative morbidity

Perioperative transient morbidity within the first 30 days after iPDT included new aphasia (n = 2), worsening of a preexisting aphasia (n = 2), new hemiparesis (n = 2), worsening of a preexisting hemiparesis (n = 1), and pulmonary embolism (n = 1). Since two patients were affected by multiple postoperative symptoms/complications, a patient-based analysis resulted in a total treatment-associated morbidity rate of 37.5% (6/16 patients). Most neurological symptoms were edema-related and improved with oral dexamethasone treatment except for one patient with a permanent new aphasia after iPDT. No long-term steroid treatment or delay of adjuvant treatment was necessary. Thus, a permanent morbidity rate of 6.3% (1/16 of iPDT patients) was recorded over the course of follow-up.

## Discussion

Adjuvant radiochemotherapy according to the EORTC/NCIC protocol has shown to improve the outcome of glioblastoma patients with a methylated *MGMT*-promoter status; however, overall survival remains limited [23]. Besides the incremental focus on biomarker profiling leading to more personalized treatment strategies, there remains an increasing need for the evaluation of more effective treatment concepts. One of these possible novel treatment concepts is iPDT for which this study provides a detailed outcome data analysis in comparison to the best available conventional treatment option. It is shown that iPDT, although requiring a careful planning procedure, is a feasible concept with an acceptable side effect profile for patients with untreated, small-sized, unresectable GBMs. A remarkable number of patients, in particular those with a methylated *MGMT*-promoter status, experienced long-term survival after iPDT. Furthermore, iPDT did not interfere with any further adjuvant treatment options.

One iPDT treated patient suffered a permanent treatment-associated morbidity. All other side effects were edema-associated and could be sufficiently treated by oral dexamethasone administration. Whenever possible, dexamethasone was waived so as to not impair potential immune-modulatory effects. No patient had to undergo long-term anti-edematous therapy and no delay of adjuvant treatment due to treatment-associated complications was recorded.

When assessing the resulting Kaplan-Meier survival curves for PFS and OS, the observed patterns for the iPDT patient cohort were found to reach a plateau indicating long-term PFS and OS for a subgroup of iPDT patients. This favorable course of disease cannot be attributed to additional adjuvant treatment since all patients, with the exception of one case merely receiving radiotherapy, only underwent initial radiochemotherapy in analogy to the EORTC/NCIC protocol with up to nine cycles of TMZ at most. Thereafter, a treatment-free period up to the first sign of tumor recurrence was initiated in all patients. Thus, the burden of adjuvant treatment could be kept to a minimum for a good proportion of these iPDT patients in the initial stages of the disease. No exceptionally aggressive recurrence treatment was noted, either. In this patient series very promising PFS and OS rates one year and two years after iPDT treatment could be recorded. Most surprisingly, a significant proportion of seven patients (43.8%) experienced a PFS > 24.0 months not linked to very aggressive salvage treatment.

Two iPDT-treated patients harbored IDH-mutant tumors and would therefore, under the current WHO classification [24], be grouped as astrocytoma, IDH mutant, CNS WHO grade 4. Possibly, this molecular profile is in part responsible for the favorable outcome in these cases, although the value of iPDT for this subgroup is not clear. IDH mutant tumors were found in the reference group in comparable numbers. Another limitation of our study cohort is the selection of small tumor volumes, so as to minimize the risk of patient harm due to space-occupying increase in edema. Whether this treatment may also benefit the many cases of larger tumors cannot be answered at this point. The patients’ excellent clinical status and prompt initiation of adjuvant therapy may have also contributed to the positive result.

A methylated *MGMT*-promoter status was associated with improved outcome parameters in both treatment groups, as is also seen in other treatment settings [7, 25]. The remarkably large survival benefit of iPDT-treated patients with positive *MGMT*-promoter methylation status, however, can most likely not only be explained by the known increased chemosensitivity by itself. Patients with an unmethylated *MGMT*-promoter profile showed outcomes similar to resected patients. Meanwhile the subgroup with methylated *MGMT*-promoter responded even better than resected patients. This response might be mediated through an immune-modulatory effect. The observed outcome improvement may thus be seen as a surrogate marker of biologically different inflammatory/immunological re-sponses to iPDT.

The precise mechanisms of tumor inactivation by iPDT, reflected in the recorded favorable PFS rate, are not entirely understood yet. Ex vivo experimental data have shown that PDT does have the ability to induce ROS in glioma cell spheroids causing consecutive necrosis [26]. It is now believed that through these events PDT does not only cause a local effect on the directly treated tumor volume but may also trigger a systemic immunological/inflammatory response which significantly contributes to the observed long-term tumor control [11, 27]. In mouse models, following PDT, an immediate infiltration of the tumor tissue by neutrophil granulocytes, mast cells, monocytes, and macrophages has been observed [28]. Preliminary ex vivo studies also suggest that iPDT impacts the adaptive immune system by increasing the cytotoxic potential of human CD8 + T-cells by alterating the cells’ mRNA and protein expression profiles [29]. These findings point to a potential iPDT-related induction of so far unknown tumor-controlling processes possibly overcoming the limitations of other local treatment concepts.

Based on our experiences, we do believe that iPDT is an appealing treatment concept for patients with newly diagnosed small-sized GBMs and deserves further evaluation in prospective clinical trials such as NCT03897491.

## Conclusion

We here show that iPDT is a promising local treatment concept for patients with newly diagnosed small-sized GBMs. Notably, despite eloquent tumor localizations, a tolerable risk profile was seen. A considerable proportion of patients, especially those with methylated *MGMT*-promoter status, experienced long-term PFS after iPDT. This might point to the induction of so far, at least in detail, unknown tumor-controlling processes, e.g. inflammatory/immunological responses. Patients treated by iPDT compared favorably in terms of PFS and OS to the cohort of patients who received the optimal conventional treatment, which is an especially impressive finding as the iPDT-treated tumors were not safely resectable. Future clinical and experimental studies should be performed to improve the understanding of the underlying cellular and serological mechanisms and, consequently, to help to identify the subset of patients most suitable for iPDT.

## Data Availability

Clinical and molecular data on all patients are anonymized and stored in local data bases secured by passwords.

## Declarations

### Additional declarations for articles in life science journals that report the results of studies involving humans and/or animals

The present study was conducted retrospectively.
